# Alternative medicine: therapeutic effects on gastric original signet ring carcinoma via ascorbate and combination with sodium alpha lipoate

**DOI:** 10.1186/s12906-022-03541-0

**Published:** 2022-03-07

**Authors:** Weiyu Chen, Lingyun Xu, Edwin Chang, Gayatri Gowrishankar, Katherine W. Ferrara, Sanjiv Sam Gambhir

**Affiliations:** 1 Department of Radiology, Stanford University School of Medicine, Stanford, CA USA; 2 Molecular Imaging Program at Stanford, Stanford University School of Medicine, Stanford, CA USA; 3 The Fourth Affiliated Hospital, Zhejiang University School of Medicine, Yiwu, 322000 Zhejiang China; 4 Canary Center at Stanford for Cancer Early Detection, Stanford University School of Medicine, Stanford, CA USA; 5 Bio-X Program at Stanford, Stanford University, Stanford, CA USA; 6 Department of Bioengineering, Stanford University, Stanford, CA USA; 7 Department of Materials Science and Engineering, Stanford University, Stanford, CA USA

**Keywords:** Ascorbate, Sodium alpha lipoate, Gastric signet ring cell carcinoma, Natural supplements, Organoid

## Abstract

**Background:**

Gastric signet ring cell carcinoma (SRCC) is an aggressive gastric adenocarcinoma with a poor prognosis when diagnosed at an advanced stage. As alternative medicine, two natural supplements (ascorbate (AA) and sodium alpha lipoate (LA)) have been shown to inhibit various cancers with mild side effects.

**Methods:**

These two natural supplements and a series of combinations (AA&LA, AA+LA and LA + AA) were incubated with non-SRCC cells (GPM-1), patient-derived gastric origin SRCC (GPM-2), gastric-origin SRCCs (HSC-39 and KATO-3), human pancreatic (MIA PaCa-2) and ovarian (SKOV-3) cells for evaluating their therapeutic effects. Moreover, these treatments were applied in 3D-cultured organoids to reveal the feasibility of these approaches for in vivo study.

**Results:**

Analyzing their antioxidant capabilities and dose-response curves, we observed that all four gastric cell lines, including three patient-derived cell lines were sensitive to ascorbate (~ 10 mM). The influence of ascorbate incubation time was studied, with a 16-h incubation found to be optimal for in vitro studies. Moreover, a simultaneous combination of AA and LA (AA&LA) did not significantly inhibit cell proliferation, while prior LA treatment increased the growth inhibition of AA therapy (LA + AA). Anti-cancer efficacy of AA was further confirmed in 3D-cultured SRCC (KATO-3) organoids.

**Conclusions:**

This study highlights the potential of AA and LA + AA in treating gastric origin SRCC, and demonstrates the influence of order in which the drugs are administered.

**Supplementary Information:**

The online version contains supplementary material available at 10.1186/s12906-022-03541-0.

## Background

Cancer resulted in ~ 600,000 deaths in the US in 2017, corresponding to 21.3% of total deaths. Among all cancers, gastric cancer is the third cause of cancer-associated death worldwide [1]. Signet ring cell carcinoma (SRCC) is one type of highly malignant gastric cancer, and has also been identified in some breast and colorectal cancers [2, 3]. Although the percentage of SRCC is relatively small across all cancer types, SRCC has a poor prognosis after surgical resection in advanced gastric cancer [3]. Occurrences of gastric cancer have decreased in the past half-century, but an increasing number of gastric cancer patients were diagnosed with SRCC [4, 5]. In addition to surgical resection, chemotherapy and radiotherapy have been widely applied in SRCC treatment, with side effects including loss of appetite and fatigue [6]. Although emerging immunotherapies, including checkpoint monoclonal antibody and chimeric antigen receptor (CAR) T-Cells provide a potential approach for SRCC therapy, the high cost limits the usage [7, 8]. A safe and affordable therapeutic strategy for SRCC is urgently needed.

In the last two decades, increasing evidence indicated that ascorbate (i.e., sodium L-ascorbate; AA), a dietary supplement, could be employed in treating cancers [9–11]. Due to the alteration of mitochondrial oxidative metabolism and reduced expression of catalases in most abnormal cells, high-dose AA acts as a pro-oxidant to induce cytotoxicity to cancer cells by generating hydrogen peroxide [9, 12]. Apoptosis can then be triggered in various carcinomas (e.g., ovarian, glioblastoma and breast cancer) when the concentration of AA reaches 3.6 to 12.3 mM [10, 11]. Notably, the addition of intravenous of vitamin C (IVC) treatment has been widely applied in treating cancer patients, and has been reported to enhance the efficacy of chemotherapy and improve the quality of life [10, 13, 14]. Another antioxidant supplement, lipoic acid (α-lipoic acid) has also been employed in treating cancer, with an IC_50_ of ~ 2.6 to 6.0 mM [15–17]. The usage of lipoic acid and its sodium form (sodium alpha lipoate; LA) can arrest the cancer cell cycle, inhibit activities of protein tyrosine phosphatase 2 (SHP2) and glucose uptake, and induce apoptosis of cancer cells [15, 16, 18]. More importantly, the combination of AA and LA was reported to trigger death of S620 cells and successfully prolong the lifespan of one patient with renal carcinoma [17, 19].

Studies of AA and LA or the combination have been conducted under varied conditions, including an incubation period ranging from 14 to 48 h [11, 15, 17]. There is not a common standard for quantifying the inhibitory effect of natural supplements. An extended duration of intravenous administration has been proposed but cannot be realistically achieved in a clinical setting [20, 21]. Therefore, it is also essential to evaluate the feasibility of any therapeutic strategy.

As opposed to traditional (i.e., two-dimensional, 2D) cell culture, a three-dimensional (3D) culture system allows patient-derived cancerous cells to grow as tumor organoids that can precisely mimic the tumor physiochemical and genetic conditions [22]. Importantly, the therapeutic efficacy of large potential set of combinatorial protocols cannot be realistically evaluated in preclinical models [23]. Tumor organoids can therefore play an important role in the evaluation of combinatorial protocols.

In the current study, we systemically evaluate the effects of AA, LA and various combinations on cell proliferation non-SRCC cells (GPM-1), patient-derived gastric origin SRCC (GPM-2), and gastric-origin SRCC lines (HSC-39 and KATO-3) [24]. The efficacy is further evaluated in human pancreatic (MIA PaCa-2) and ovarian (SKOV-3) cell lines.

## Methods

### Reagents and preparation

Sodium L-ascorbate and alpha-lipoic acid were obtained from Sigma-Aldrich. The catalase activity assay was purchased from Abcam. The catalase antibody was obtained from Santa Cruz. The PrestoBlue™ BCA protein assay kit cell viability reagent and calcein, AM, cell-permeant dye were obtained from ThermoFisher. Other reagents used in the current study have been purchased from ThermoFisher.

Ascorbate (AA) and sodium alpha lipoate (LA, sodium salt of alpha-lipoic acid) were prepared freshly for each assay. The preparation of sodium alpha lipoate followed previous reports [25]. Briefly, lipoic acid and sodium bicarbonate were mixed equally and the solution was lyophilized to recover sodium alpha lipoate. The concentration of solution was determined by the UV absorbance at 330 nm.

### Cell culture

SRCC gastric (KATO-III) cancer cell lines were obtained from the American Type Culture Collection (ATCC). The patient-derived gastric cancer cell lines including SRCC (GPM-2 and HSC-39) and non-SRCC (GPM-1) were gifted by Prof. Hayao Nakanishi, Aichi Cancer Center Research Institute. Human pancreatic (MIA PaCa-2) and ovarian (SKOV-3) cancer cells lines that are sensitive and resistant to ascorbate respectively, were purchased from ATCC.

For 2D culture, cells cultured in standard Iscove’s Modified Dulbecco’s Medium (IMDM), Dulbecco’s modified Eagle’s medium (DMEM) or RPMI-1640 were supplemented with 10% fetal bovine serum (FBS). For 3D culture, KATO-3 cell suspension was mixed with collagen gel solution (cellmatrix type I collagen, 10 x IMDM, 0.005 N NaOH and 20 mM HEPES). Then, cells in a 20 ul gel were added to each well (96-well plate) and incubated at 37 °C for 30 mins. Cells were cultured in standard Iscove’s Modified Dulbecco’s Medium (IMDM) supplemented with 10% fetal bovine serum (FBS).

### Western blot

Cells were harvested and suspended in lysis buffer (Abcam). Total protein concentrations were detected first by using the BCA protein assay kit (Thermo Fisher Scientific). Samples were loaded and separated by SDS-PAGE gel via electrophoresis. Antibodies including anti-catalase and GAPDH (Bioleagend) were further applied to examine the expression of catalase among different cell types. The membrane obtained was scanned on a ChemiDoc imaging system (Bio-rad).

### Antioxidant activity assay

Briefly, cells (> 2 × 10^6^ cells) were harvested and washed with PBS twice and finally suspended in the same volume of lysis buffer (Abcam). A volume of cell lysis buffer was added into wells containing H_2_O_2_ and incubated for 30 mins, with (A_HC_) or without stop solution (A_sample_). After termination of the reaction, HRP and OxiRed probe were added into wells and developed for 10 mins at 25 °C. The absorbance at OD = 570 nm was detected on a multi-channel fluorescent microplate reader (Tecan 800). The antioxidant activity (nmol/min/mg) was calculated by the formulation below:

$$\mathrm{Antioxidant}\ \mathrm{activity}=\frac{B}{30\times V}/C$$

B = amount of H_2_O_2_ that was calculated from (A_HC_-A_sample_) from the standard curve;

C = protein concentration of the sample (BCA assays).

### In vitro studies of gastric SRCC and non-SRCC cancer cell lines

1. Assessment of the sensitivity of PrestoBlue™:

KATO-3/MIA PaCa-2 cells for 2D culture or KATO-3 cells suspension mixed with collagen for 3D culture were seeded in 96-well black plate with transparent bottom (Corning) at a final concentration at cell density from 10 to 10^4^ cells per well overnight. The cells were then incubated and detected via PrestoBlue™ assays.

2. Assessment of AA and LA treatments:

Cells were seeded in a 96-well plate (Corning) at a final concentration at 1 × 10^4^ cells/well overnight. The culture medium was replaced by freshly-prepared medium containing different concentrations of AA, LA or both natural supplements as below:

For the optimization of incubation time, cells were incubated with reagents (AA) for 1, 16 or 72 h, respectively. Then cells were washed and further cultured in fresh medium for the remaining incubation period (72 h);

For simultaneously-combined treatments (AA&LA), cells were treated with AA and LA simultaneously for 16 h. Then cells were washed and further cultured in fresh medium for the remaining incubation period (72 h);

For protocols mimicking a realistic intravenous protocol (clinically viable protocol), cells were treated as follows:

- AA or LA: single supplement for 3 h;
- LA + AA: incubated with LA, then media and AA each for 3 h, respectively;
- AA+LA: incubated with AA, then media and LA each for 3 h, respectively.

Cells were then washed and further cultured in fresh medium for the remaining incubation period (72 h). After treatment, cell viability was detected by PrestoBlue™ assays. For 3D-cultured cells, the supernatant after incubation was transferred to a 96-well plate for reading.

We define the “inhibition percentage” as the decrease of living cells as determined via PrestoBlue™ fluoresence intensity. The minimum threshold of inhibition percentage (MTI %) was the average inhibition percentage calculated from the lowest three concentrations studied and is calculated by 100%-(fluorescence intensity of treated cells/ fluorescence intensity of untreated cells)*100%.

3. For investigation of AA on 3D-cultured KATO-3 cells:

KATO-3 cells were seeded in collagen for 3D culture at cell density of 10^4^ cells per well overnight. Cells were incubated with AA for 3 h and then cultured for 69 h in fresh medium. Cell viability was detected via PrestoBlue™ assays.

### Celigo imaging

Following the treatments above, cells were stained by calcein AM, cell-permeant dye for 20 mins (at dilution 1:3000). The fluorescence and bright-field images of each well were then acquired on a Celigo image cytometer (Nexcelom Bioscience, Lawrence, MA, USA).

### Statistical analysis

All data were analyzed on PRISM 8 (GraphPad) by a unpaired 2-tailed student’s t-test or two-way ANOVA with multiple hypothesis correction (Tukey’s test). *P* values < 0.05 were considered statistically significant, * *p* < 0.05, ** *p* < 0.01, ** *p* < 0.001, **** *p* < 0.0001.

## Results

### SRCC has finite catalase expression and antioxidant capabilities

**Fig. 1:**
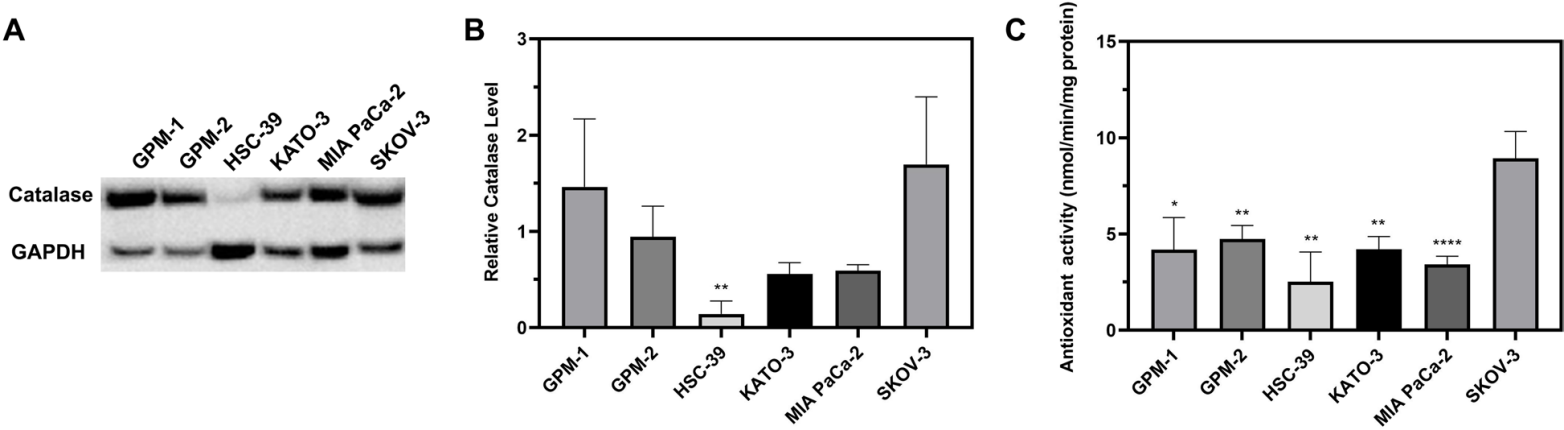
Antioxidant activity among non-SRCC cells (GPM-1), patient-derived gastric origin SRCC (GPM-2), gastric-origin SRCCs (HSC-39 and KATO-3) and human pancreatic (MIA PaCa-2) and ovarian (SKOV-3) lines. The catalase expression among different cells, which was determined by **A** western-blot and **B** relative catalase expression levels. **C** Antioxidant activities of different cells via exogenous H_2_O_2_ scavenge. Data were analyzed by a unpaired 2-tailed student’s t-test. Significance is reported for the difference between the labeled cells and SKOV-3 cells. *P* values < 0.05 were considered statistically significant (* *p* < 0.05, ** *p* < 0.01, ** *p* < 0.001, **** *p* < 0.0001)

As the cytotoxicity of AA is related to the cellular antioxidant capability, the expression of catalase was examined among various gastric original carcinoma cells. As shown in Fig. 1A and B, all SRCC lines, including GPM-2, HSC-39 and KATO-3, had limited catalase expression. More specifically, HSC-39 cells expressed the lowest level of relative catalase (0.142 ± 0.136), with a significant difference with respect to the ROS-resistant cell line SKOV-3 (*P* = 0.0063) [26]. Notably, these SRCC lines produced a comparable level of catalase to the MIA PACA-2 line that is sensitive to AA [9]. The non-SRCC line GPM-1 produced a larger amount of catalase; the amount is comparable to the SKOV-3 line.

In addition to catalase, superoxide dismutase (SOD)-1 and − 2 also exhibit antioxidant effects. The total antioxidant enzymes among GPM-1, MIA PACA-2 and other SRCC lines were lower than those in SKOV-3, with *P* values ranging from < 0.0001 to 0.0107 (Fig. 1C), which clearly indicate the potential for AA treatment to impact these cells.

### Ascorbate-mediated cytotoxicity on SRCC was enhanced by extended incubation time

**Fig. 2:**
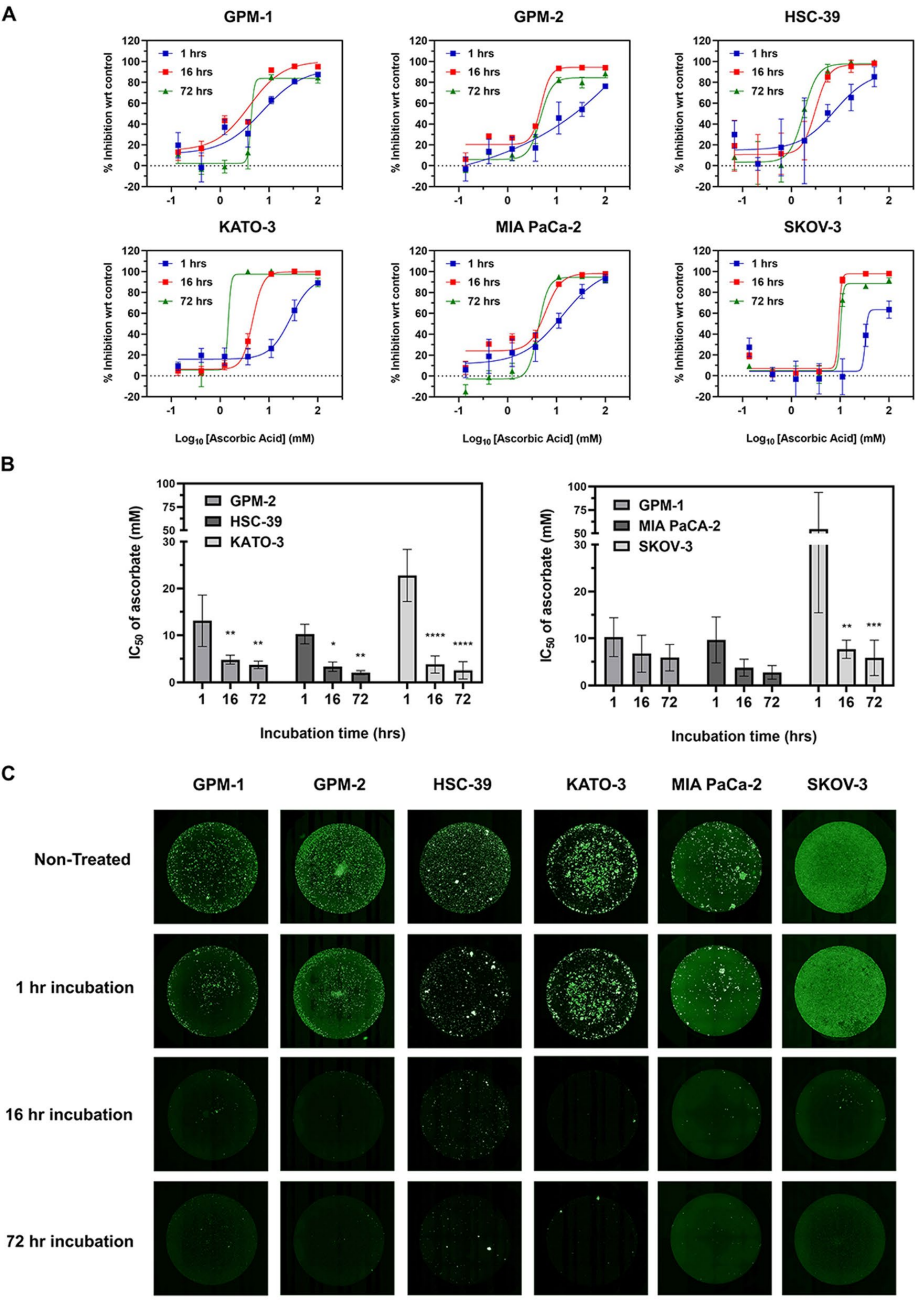
Extending the incubation time enhanced the IC_50_ for all SRCC cell lines. Influence of incubation time with AA on non-SRCC cells (GPM-1), patient-derived gastric origin SRCC (GPM-2), gastric-origin SRCCs (HSC-39 and KATO-3), and human pancreatic (MIA PaCa-2) and ovarian (SKOV-3) lines. **A** Inhibition curves of A among various cells after incubations of 1, 16 and 72 h, respectively. **B** IC_50_ of AA after 1, 16 and 72 h on SRCC (left) and non-SRCC (right) (*n* = 3). Data were analyzed by two-way ANOVA. *P* values < 0.05 were considered statistically significant. Significance is reported for the difference between the labeled groups (i.e., 16- or 72-h) and 1-h treated group (* *p* < 0.05, ** *p* < 0.01, ** *p* < 0.001, **** *p* < 0.0001). **C** Cell viability after incubation with AA. After treatment, cells were stained with calcein and scanned on a Celigo imaging cytometer

The anti-cancer effect of AA has been verified in various cancer cells, but treatment conditions (i.e., incubation time of AA) were varied among studies, which leads to inconsistent IC_50_ values. To examine the importance of treatment time, SRCC was incubated with AA for different durations (1, 16 and 72-h incubation). PrestoBlue™, a fluorescent cell viability reagent was selected for current studies, which demonstrated high sensitivity for detecting small amounts (as low as ten cells) of living KATO-3 and MIA PaCa-2 cells (Supplementary Fig. S1). As shown in Fig. 2A, inhibition resulting from AA was apparent after 16-h incubation, while the inhibitory effect of AA was rarely apparent at 1-h post-treatment. Specifically, the IC_50_ among non-SRCC lines was not significantly decreased by increased incubation time (Fig. 2B and C). In comparison, the IC_50_ of AA in treating SRCC was effectively reduced via an extended incubation (i.e., 16-h), showing the efficiency of AA in treating SRCC. The IC_50_ was saturated near 16 h, with slight changes as the AA treatment time increased from 16 to 72 h (Fig. 2 and Table 1). These findings also suggest that a 16-h incubation is optimal for determining the IC_50_ resulting from AA treatment.

**Table 1:** IC_50_ (mM) of cells after 1, 16 and 72-h treatments with ascorbate

Cancer lines		Patient-derived Non-SRCC	Patient-derived SRCC	SRCC cell lines		Pancreatic	Ovarian
Treatment (hr)	Time of assessment (hr)	GPM-1	GPM-2	HSC-39	KATO-3	MIA PaCA-2	SKOV-3
1	72	10.3 ± 4.15	13.1 ± 5.48	10.2 ± 2.09	22.9 ± 5.57	9.67 ± 4.90	54.6 ± 39.2
16	72	6.75 ± 3.92	4.79 ± 0.96	3.34 ± 0.97	3.78 ± 1.80	3.76 ± 1.78	7.67 ± 1.93
72	72	5.88 ± 2.80	3.70 ± 0.79	2.06 ± 0.44	2.55 ± 1.83	2.76 ± 1.42	5.85 ± 3.75

### Treatments with LA, AA and combinations inhibit SRCC

**Fig. 3:**
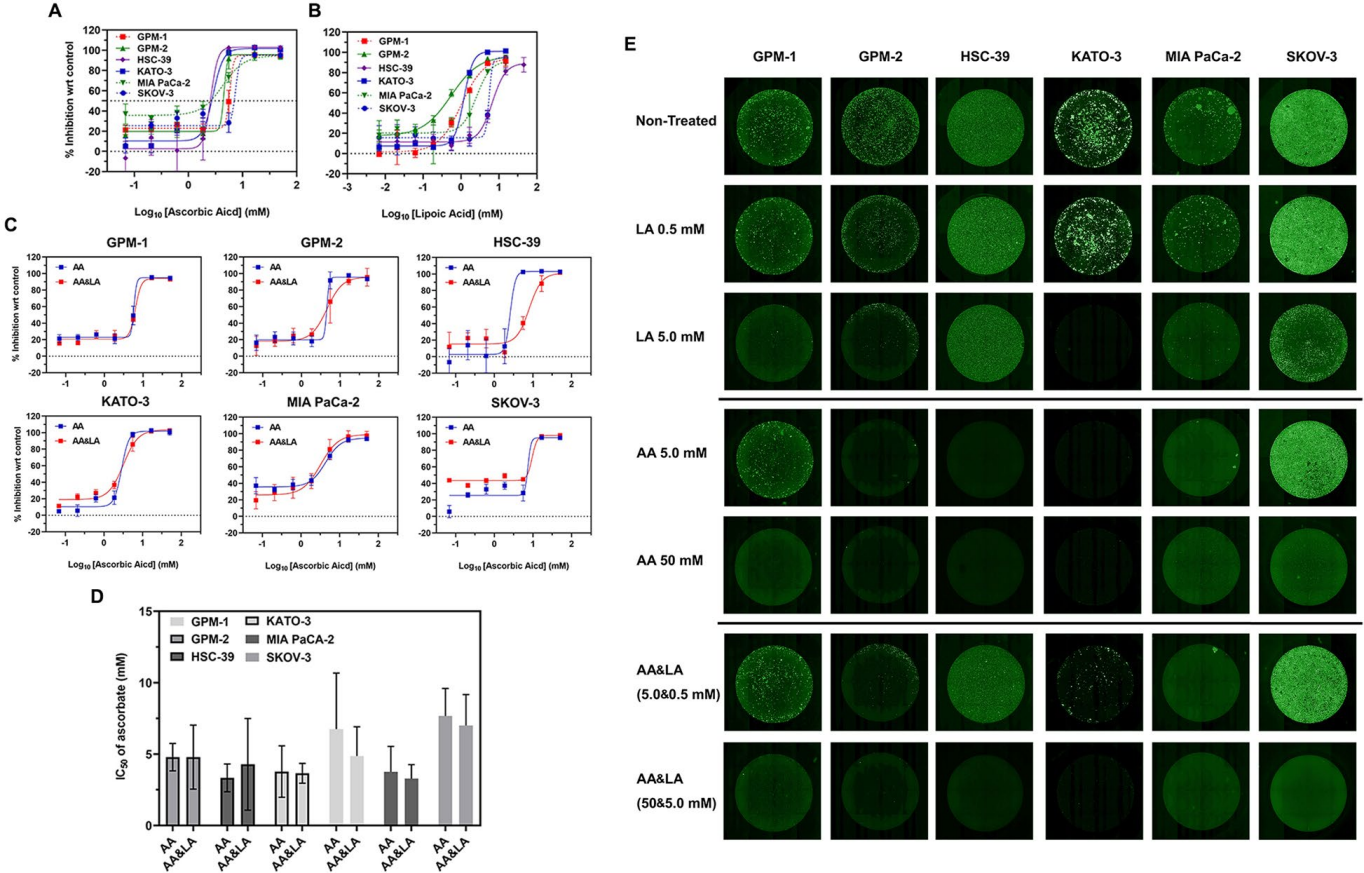
Separate and simultaneous treatment of AA and LA on non-SRCC cells (GPM-1), patient-derived gastric origin SRCC (GPM-2), gastric-origin SRCCs (HSC-39 and KATO-3) and human pancreatic (MIA PaCa-2) and ovarian (SKOV-3) lines. **A** Inhibition curves of AA after 16-h incubation. **B** Inhibition curves of LA after an incubation of 16 h. **C** 16-h inhibition curves of AA & LA (simultaneously) on various cells. **D** IC_50_ of all cells after AA and AA&LA treatments. **E** Viability of cells after incubations with AA, LA or combinations, respectively. Cells after treatments were stained with calcein and scanned on a Celigo imaging cytometer

Similar to AA (Fig. 3A), LA also demonstrated cytotoxicity and inhibited the growth of SRCC, with values of IC_50_ ranging from 0.69 to 2.80 mM (Fig. 3B and Supplementary Fig. S2). Necrosis was resulted from LA incubation. For example, on HSC-39 cells 5 mM AA or 1.6 mM LA induced obvious morphological changes. Similar effects were observed with non-SRCC (GPM-1) and SRCC (GPM-2) cells (Supplementary Fig. S3).

Since simultaneous treatment with AA and LA (with a ratio of AA: LA of 10:1) was reported to improve the therapeutic efficacy compared with a single treatment of AA, the influence of combinations was further examined on SRCC. However, the simultaneous combination of AA and LA (AA&LA) did not enhance SRCC killing in terms of IC_50_ or over-all inhibition rate (Fig. 3C-E and Table 2). Moreover, the addition of LA suppressed the effect of AA in specific cell lines although these changes were not significant. For instance, the IC_50_ of AA on HSC-39 increased from 3.34 ± 0.97 mM (single treatment of AA) to 4.29 ± 3.21 mM in AA and LA simultaneously-combined therapy.

**Table 2:** IC_50_ (mM) of cells after 16-h treatments of sodium alpha lipoate (LA), ascorbate (AA) and the simultaneous combination (AA&LA)

Cancer lines		Patient-derived Non-SRCC	Patient-derived SRCC	SRCC cell lines		Pancreatic	Ovarian
Reagents	Treatment/Assessment (hr)	GPM-1	GPM-2	HSC-39	KATO-3	MIA PaCA-2	SKOV-3
LA	16/72	1.29 ± 0.36	0.69 ± 0.20	2.80 ± 2.20	1.46 ± 0.48	2.00 ± 0.70	4.13 ± 0.55
AA	16/72	6.75 ± 3.92	4.79 ± 0.96	3.34 ± 0.97	3.78 ± 1.80	3.76 ± 1.78	7.67 ± 1.93
AA&LA	16/72	4.87 ± 2.04	4.79 ± 2.42	4.29 ± 3.21	3.66 ± 0.70	3.29 ± 0.98	7.00 ± 2.17

For AA&LA, cells were treated with AA and LA at a ratio of AA: LA of 10:1

### Dosing schedules for AA+LA impact the therapeutic effect on SRCC for limited incubation times

**Fig. 4:**
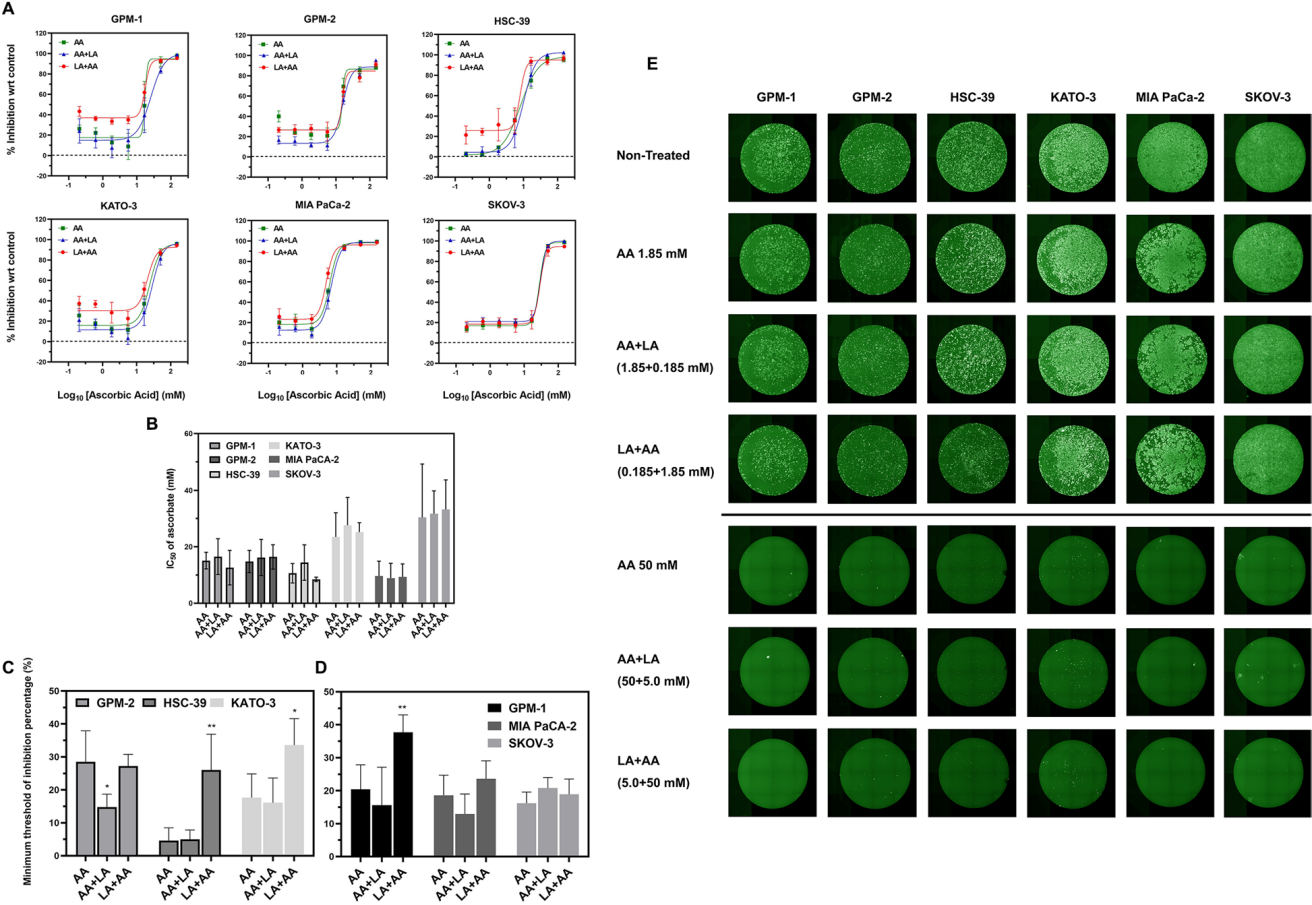
Effect of 3-h incubation with AA and LA on non-SRCC cells (GPM-1), patient-derived gastric origin SRCC (GPM-2), gastric-origin SRCCs (HSC-39 and KATO-3) and human pancreatic (MIA PaCa-2) and ovarian (SKOV-3) lines. **A** Inhibition curves after: 3-h AA, 3-h AA + 3-h medium + 3-h LA (denoted as AA+LA) or 3-h LA + 3-h medium + 3-h AA (denoted as LA + AA). **B** IC_50_ of all cells after 3-h treatments of AA, AA+LA or LA + AA. Minimum threshold of inhibition percentage after: 3-h AA, 3-h AA+LA or 3-h LA + AA in **C** SRCC and **D** non-SRCC (*n* = 3). Significance is reported for the difference (* *p* < 0.05, ** *p* < 0.01) between labeled groups and single treatment (e.g., AA). The values are summarized from inhibition percentage of the lowest three concentrations (e.g., AA = 0.206, 0.617 and 1.852 mM) used among treatments (e.g., AA, AA+LA and LA + AA). All data were analyzed by a two-way ANOVA. **E** Viability of cells after incubations with three different treatments. After treatments, cells were stained with calcein and scanned on a Celigo imaging cytometer

As shown previously, extended incubation (16 h) of these natural agents is needed for growth inhibition of SRCC. However, the pharmacokinetics of AA and LA in a clinical setting (with a limited (3–4 h) exposure time) is dramatically different from that with extended cell incubation. Therefore, clinically-relevant incubation was performed to investigate the combined therapeutic effect and the influence of incubation time. As expected, the IC_50_ of AA or other combinations obtained from clinically-relevant (3-h incubation/infusion) assays was higher than that calculated previously (Fig. 4A-B and Table 3). Short-term incubation with LA exhibited only mild cytotoxicity on cancerous cells (Supplementary Fig. S4). Notably, simultaneously-combined AA and LA (e.g., 3-h AA&LA incubation) showed a limited inhibitory effect, while staggered treatments including LA + AA (3-h LA incubation, then 3-h media, final 3-h AA treatment) and AA+LA (3-h AA incubation, then 3-h media, final 3-h LA treatment) demonstrated different inhibition kinetics (Fig. 4C-E). Specifically, the addition of LA prior to AA incubation (i.e., the LA + AA approach) increased the minimal threshold of inhibition percentage (MTI%) significantly for SRCC lines (HSC-39 and KATO-3) and GPM-1(non-SRCC) compared to the simultaneous addition of LA and AA (Fig. 4C and D). For example, for LA + AA the MTI% was increased ~ 5 times in HSC-39 cells compared with AA treatment. In contrast, the MTI% was reduced when AA was applied before LA (AA+LA). For example, the MTI% for GPM-2 decreased by 13.7% after AA+LA treatment in comparison with a single treatment of AA.

**Table 3:** IC_50_ (mM) and MTI% (%) of cell lines after 3-h treatments with ascorbate (AA) and combination where either AA was administered first (AA+LA) or LA was administered first (LA + AA). MTI%: Minimum threshold of inhibition percentage (calculated from the average of lowest three concentrations)

	Cancer lines		Patient-derived Non-SRCC	Patient-derived SRCC	SRCC cell lines		Pancreatic	Ovarian
Assays	Reagents	Treatment/Assessment time (hr)	GPM-1	GPM-2	HSC-39	KATO-3	MIA PaCA-2	SKOV-3
IC_50_ (mM)	AA	3/72	15.1 ± 2.95	14.8 ± 3.92	10.6 ± 3.43	23.5 ± 8.56	9.62 ± 5.27	30.4 ± 18.8
	AA+LA	3 + 3/72	16.5 ± 6.34	16.2 ± 6.41	14.4 ± 6.28	27.6 ± 9.88	8.88 ± 5.25	31.7 ± 8.11
	LA + AA	3 + 3/72	12.6 ± 6.11	16.4 ± 4.32	8.44 ± 0.79	25.2 ± 3.33	9.33 ± 4.58	33.2 ± 10.5
MTI% (%)	AA	N/A	20.4 ± 7.44	28.5 ± 9.42	4.61 ± 3.84	17.6 ± 7.24	18.6 ± 6.09	16.2 ± 3.39
	AA+LA	N/A	15.6 ± 11.5	14.8 ± 3.90	4.97 ± 2.84	16.1 ± 7.49	12.9 ± 6.08	20.8 ± 3.21
	LA + AA	N/A	37.7 ± 5.29	27.2 ± 3.56	26.0 ± 10.9	33.6 ± 8.01	23.6 ± 5.44	18.9 ± 4.62

For AA+LA: cells were incubated with AA, media and LA for 3 h, respectively. IC_50_ of AA was analyzed after a total incubation of 72 h (keep a ratio of AA: LA at 10:1). Results were shown by IC_50_ of ascorbate (AA)

For LA + AA: cells were incubated with LA, media and AA for 3 h, respectively. IC_50_ of AA were analyzed after a total incubation 72 h (keep a ratio of AA: LA at 10:1). Results were shown by IC_50_ of ascorbate (AA)

### Therapeutic effect of AA on 3D-cultured SRCC

**Fig. 5:**
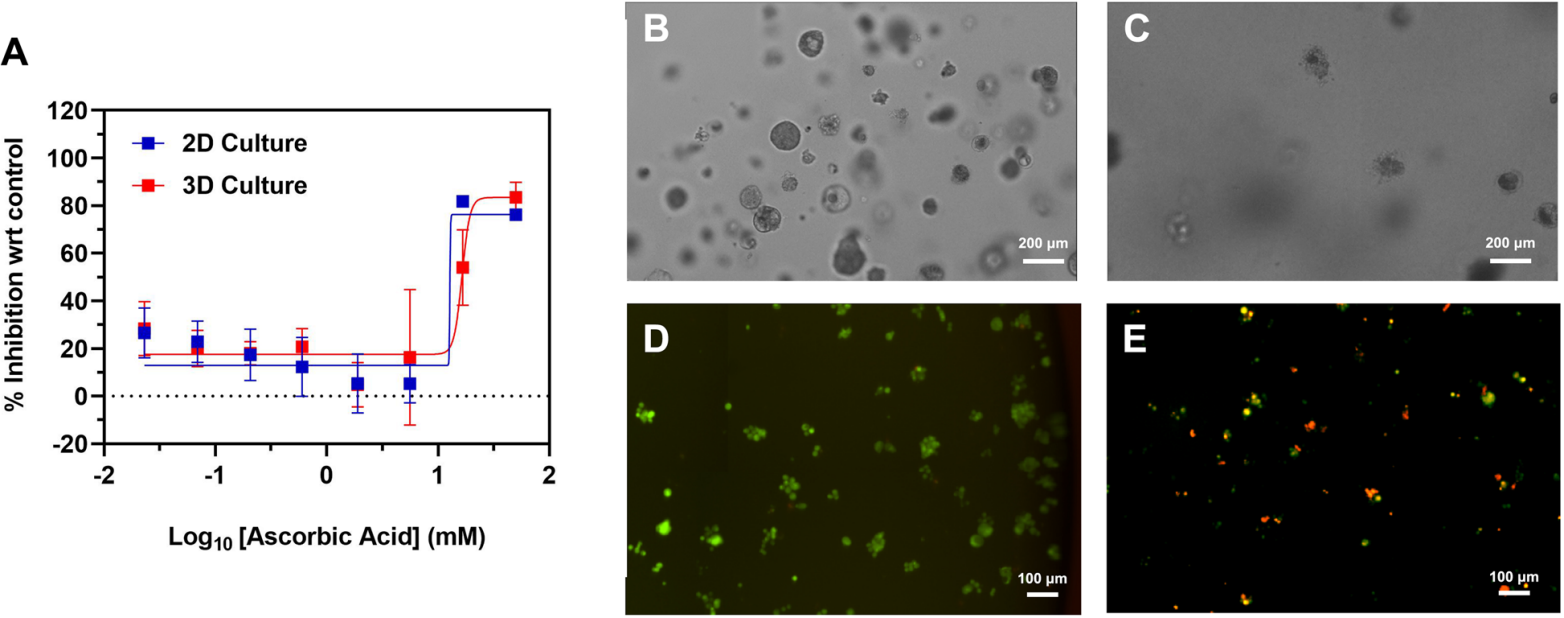
Effect of AA on 3D-cultured SRCC cells. **A** Inhibition curves of 2D- and 3D-cultured KATO-3 cells after 3-h AA treatment. Optical images of **B** KATO-3 and **C** KATO-3 after 3-h AA incubation (25 mM) in collagen. Calcein AM staining on 3D-cultured KATO-3, **D** cells only and **E** 3-h AA incubation (25 mM). Scale bars are 100 and 200 um

Given that physicochemical conditions within malignant tissue are complex as compared with traditional cell culture, the feasibility of AA treatment for SRCC was further evaluated in 3D-cultured organoids. As seen in Supplementary Fig. S5, KATO-3 cells grew into cancerous organoids that could be detected via cell viability reagents. Although a 3D structure reduces the surface for interaction, similar AA dose-dependent curves were observed in treating KATO-3 cells growth in 2D and 3D, with an IC_50_ of 12.77 to16.41 mM (Fig. 5A). Specifically, the cell necrosis and breakdown of organoids were evident after AA incubation (25 mM), indicating the availability of AA for SRCC therapy (Fig. 5B-E).

## Discussion

The use of natural supplements as alternative cancer therapies has increased, as a result of the reduced side effects compared with other treatments [27]. In our current study, two candidates, ascorbate (AA) and sodium alpha lipoate (LA) were employed for treating gastric-origin SRCC, a type of highly aggressive cancer and non-SRCC cancers. The cytotoxicity of the natural supplements was systemically investigated, including the influence of incubation time and administration approach. Our findings indicate that both natural supplements inhibit the growth of cancerous cell lines and patient-derived SRCC and non-SRCC cells, and the 16-h incubation is the optimal duration for determining the IC_50_. We varied the drug input order and assessed the impact on cytotoxicity. The therapeutic effect of AA was further confirmed in 3D-culture SRCC (KATO-3) organoids.

In general, the antioxidant enzyme concentration is reduced in cancerous compared with normal cells [9]. Short-term exposure of AA can increase the oxidant pressure and inhibit cancer cell proliferation, with a typical IC_50_ below 10 mM (75% of 43 cancer cells) [28]. More importantly, the inhibition efficiency of AA is related to the cellular expression of antioxidant enzymes, as previously shown in MIA-PaCa-2 [9]. We found that all three SRCC and GPM-1 (non-SRCC) exhibited similar levels of antioxidant capabilities in a dose-dependent fashion (Fig. 1C). The results indicate the feasibility of AA therapy for treating SRCC.

In vitro assays have been widely used to determine the dose-response curves and IC_50_ among drug candidates. However, the efficacy is impacted by parameters such as the drug incubation time (which must be optimized). Ascorbate, as a pro-oxidant, interacts with singlet oxygen and generates hydrogen peroxide, typically considered to be a rapid reaction that should not be affected by incubation time. Nevertheless, the dose-response curves were impacted by changes in the exposure time, for instance, the IC_50_ of SKOV-3 was reduced from 79.4 to < 3 mM after extending the incubating time from 16 to 48 h [10, 11]. Similarly, our findings also suggest that extended exposure to AA could greatly increase the cytotoxicity on SRCC, indicating the therapeutic effect of AA for dealing SRCC (Fig. 2). Notably, a 16-h incubation was most effective in demonstrating treatment-related differences, while 1- and 72-h reaction times induced insufficient or saturated effects (Table 1). More importantly, the results show, for the first time, the influence of AA incubation time on the same time-scale, and provided the opportunity to optimize the in vitro strategy for studying the anti-cancer effect of AA in the future.

The pharmacokinetics of drugs in a clinical setting differs dramatically from in vitro studies. Although the peak concentration of AA and LA can reach ~ 21.8 mM in mice (60 g) and 14 ~ 30 mM in human (25 ~ 100 g) after IVC, circulation is less than 4 h in most cases [21]. Therefore, study design geared to mimic the plasma bioavailability was essential to examine the feasibility of potential for translation to patients. A clinically-feasible approach (i.e., 3-h incubation) was applied to evaluate actual availability of drugs in treating patients. Although the IC_50_ was higher than achieved after 16-h incubation, an IC_50_ of AA for HSC-39, GPM-2 and GPM-1 was achievable via the 3-h incubation, either for small animals or humans (Table 3). Moreover, AA effectively prohibited growth and induced necrosis of SRCC (KATO-3) organoids that better recapitulate the architectures of malignant tissues. In addition to 2D cell lines, evaluation in organoids further verifies the applicability of the proposed strategies for in vivo studies.

Alternatively, LA triggers the death of cancer cells by alternative and less cytotoxic mechanisms, for instance, alteration of the Warburg effect [15]. In previous studies, the LA IC_50_ for cancerous cells ranged between 2.6 and 6.0 mM for a 48-h incubation, while the current IC_50_ in the four SRCC/Non-SRCC cell lines ranged from 0.7 to 2.8 mM after 16-h incubation (Fig. 3B and Table 2) [15–17]. As a supporting agent, LA was shown to boost the therapeutic efficacy for simultaneous application of AA and LA [17]. However, the synergistic effect of simultaneous administration was small in the current study (Fig. 3 and Table 2).

Interestingly, the input order greatly affected the cytotoxicity of treatments (Fig. 4 and Table 3). More specifically, the addition of LA prior to AA efficiently enhanced the inhibition during staggered therapy, while the opposite order arrested and even suppressed the cytotoxicity (Table 3). Most importantly, when LA was administered before AA, growth inhibition with a short incubation and low dose (defined as MTI%) increased on SRCC lines and GMP1(non-SRCC) (Fig. 4C and D). Previously, incubation of LA was shown to reduce the expression of various antioxidant enzymes (e.g., superoxide dismutase (SOD)-1/2 and catalase) on cancer cells [29]. The pre-treatment with LA can potentially sensitize cancer cells and enhance the anti-cancer effect mediated by AA (Fig. 4). In contrast, the simultaneous or subsequent incubation of LA can suppress this effect by acting as an antioxidant reagent (Fig. S3 and 4). On the other hand, the harsh growth environment triggered by AA may also force cancer cells to change or slow down their metabolism, and could reduce the efficacy of LA (i.e.*,* inhibition of Warburg effect). Therefore, LA alone was applied and increased MTI% compared with other combinations. Notably, AA-based therapies showed similar patterns in treating both 2D- and 3D-culture SRCC (Fig. 5A). These findings have provided a hypothesis as to how the drug input order impacts the therapeutic effect of AA and LA. As a result, treatment with LA prior to AA enhanced anti-cancer efficacy in models of gastric cancer, especially for SRCC (Fig. 4). Based on the success of LA and AA combined therapy, pre-treatment with LA may improve and provide a higher therapeutic effect in future clinical applications [19].

In complementary and alternate medicine, IVC has been successfully applied in preclinical and clinical trials, including combinations of IVC/gemcitabine/erlotinib or IVC/chemo-radiation [30–32]. Desirable outcomes were achieved with rare side effects observed in most cases. Thus, the LA + AA- or AA-combined treatments have potential for treating oxidant-sensitive gastric cancer (i.e.*,* SRCC). Notably, AA incubation could trigger H_2_O_2_-mediated disruption of Fe metabolism [30]. Additionally, the combination of AA and an anti-PD-1 antibody could enhance CD8^+^ T lymphocyte functionality [33]. The combination of a ferroptosis inducer (i.e., Erastin), anti-PD-1 antibody and AA/LA + AA may also be considered for gastric cancer therapy.

## Conclusion

In conclusion, we systemically investigated and illustrated the combined anti-cancer effect of two natural supplements, AA and LA in treating gastric original SRCC/non-SRCC grown in traditional 2D culture and 3D-growth organoids. More importantly, the influence of drug-input order was described for the first time for combined therapy with LA and AA. These findings suggest that combined AA and LA treatment could provide an alternative medicine for treating SRCC and are potentially complementary in combined therapy.

## Supplementary Information

**Additional file 1.**

## Data Availability

All data associated with current study have been included in the article or uploaded as supplementary information.

## Abbreviations

SRCC: Gastric signet ring cell carcinoma
AA: Ascorbate
LA: Sodium alpha lipoate
CAR-T: Chimeric antigen receptor T-Cells
IVC: Intravenous of vitamin C
SHP2: Protein tyrosine phosphatase 2
2D: Two-dimensional
3D: Three-dimensional
SOD: Superoxide dismutase
MTI %: Minimum threshold of inhibition percentage
