# Supplementary material for: Alternative medicine: therapeutic effects on gastric original signet ring carcinoma via ascorbate and combination with sodium alpha lipoate
Source: BMC Complement Med Ther. 2022 Mar 7;22:58. doi: 10.1186/s12906-022-03541-0 (PMC8903574; doi:10.1186/s12906-022-03541-0)
Supplement: Supplementary file 1 — Additional file 1. [file 12906_2022_3541_MOESM1_ESM.docx]

**Supplementary information**

**Alternative medicine: therapeutic effects on gastric original signet ring carcinoma via ascorbate and combination with sodium alpha lipoate**

Weiyu Chen^1,2,3^, Lingyun Xu^1,4^, Edwin Chang^1,4^, Gayatri Gowrishankar^1,2^, Katherine W. Ferrara^1,2,7*^, Sanjiv Sam Gambhir†^1,2,4,5,6,7^

1. Department of Radiology, Stanford University School of Medicine, Stanford, CA, USA

2. Molecular Imaging Program at Stanford, Stanford University School of Medicine, Stanford, CA, USA

3. The Fourth Affiliated Hospital, Zhejiang University School of Medicine, Yiwu, Zhejiang, 322000, China

4. Canary Center at Stanford for Cancer Early Detection, Stanford University School of Medicine, Stanford, CA, USA

5. Department of Bioengineering, Stanford University, Stanford, CA, USA

6. Department of Materials Science and Engineering, Stanford University, Stanford, CA, USA

7. Bio-X Program at Stanford, Stanford University, Stanford, CA, USA

* To whom correspondence should be addressed

Weiyu Chen, Ph.D. Email: [weiyuchen@zju.edu.cn](mailto:weiyuchen@zju.edu.cn)

Katherine Ferrara, Ph.D. Email: [kwferrar@stanford.edu](mailto:kwferrar@stanford.edu)

2. Department of Radiology, Stanford University, CA, USA;

5. Molecular Imaging Program at Stanford, Stanford University School of Medicine, Stanford, CA, USA

**Supplementary Figures**


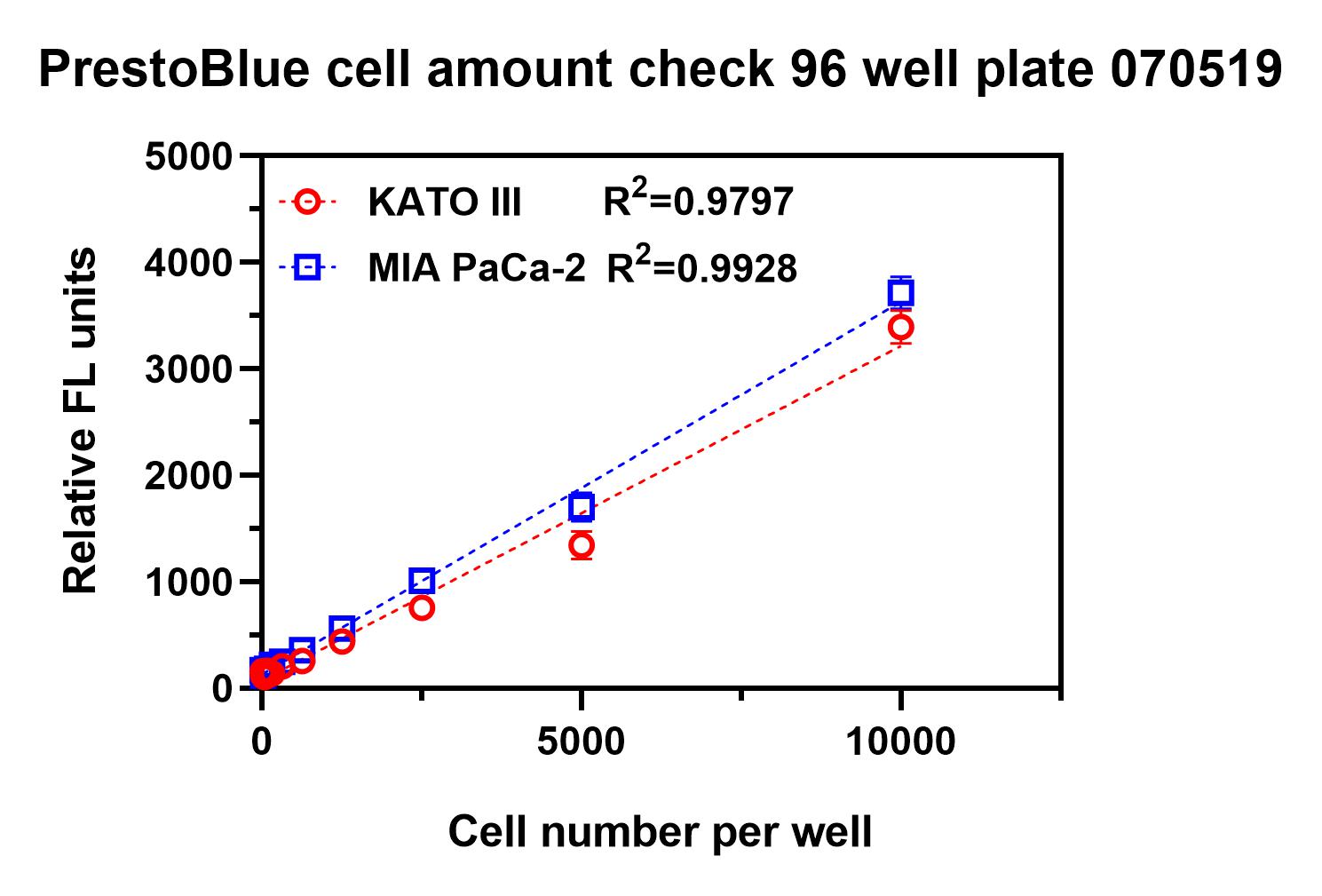


**Supplementary Fig. S1** The relationship between cell number and fluorescence signal generated by PrestoBlue™.


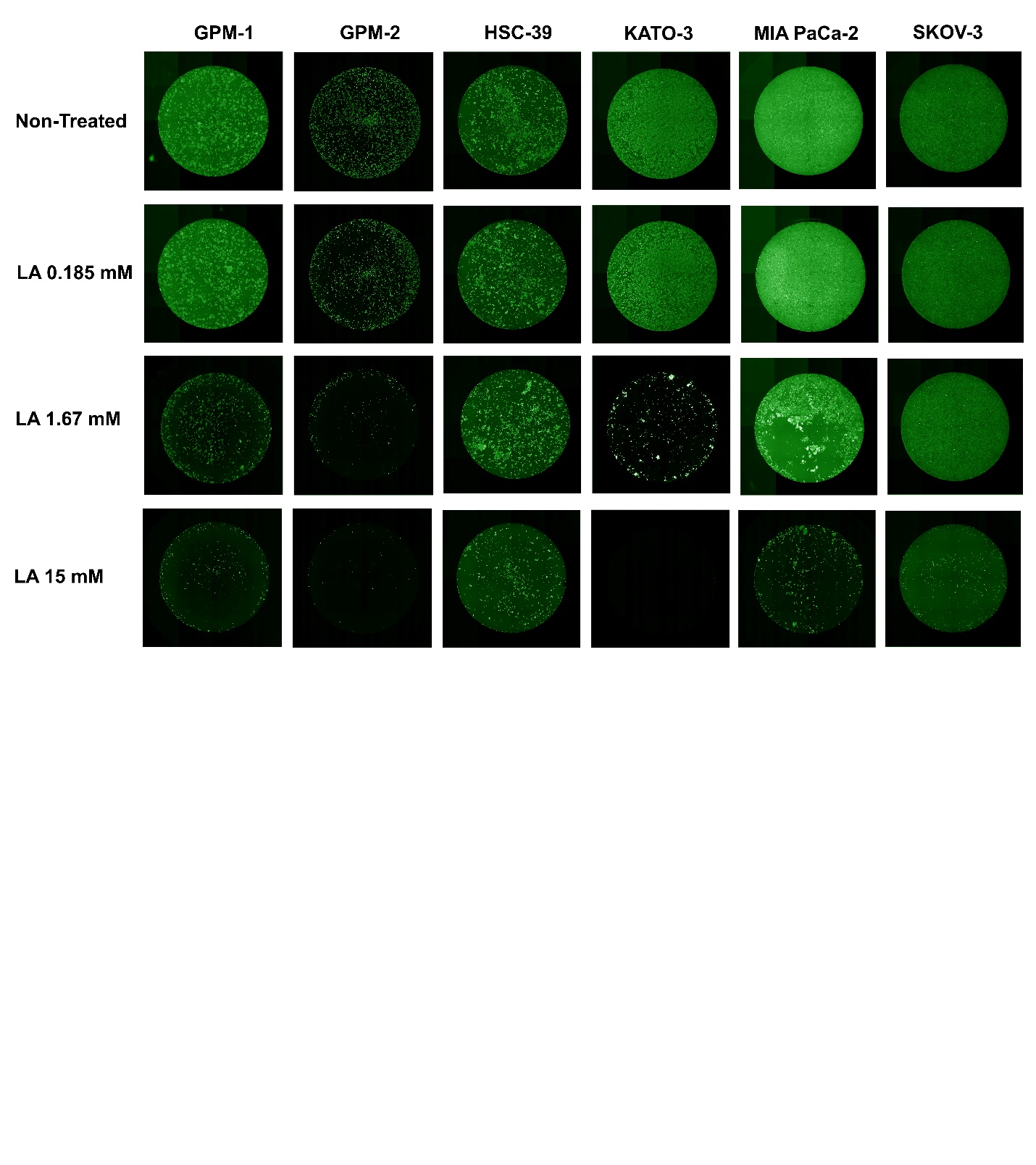


**Supplementary Fig. S2** 16-hour treatment with LA on non-SRCC cells (GPM-1), patient-derived gastric origin SRCC (GPM-2), gastric-origin SRCCs (HSC-39 and KATO-3) and human pancreatic (MIA PaCa-2) and ovarian (SKOV-3) lines. After treatment, cells were stained with calcein and scanned on a Celigo imaging cytometer.


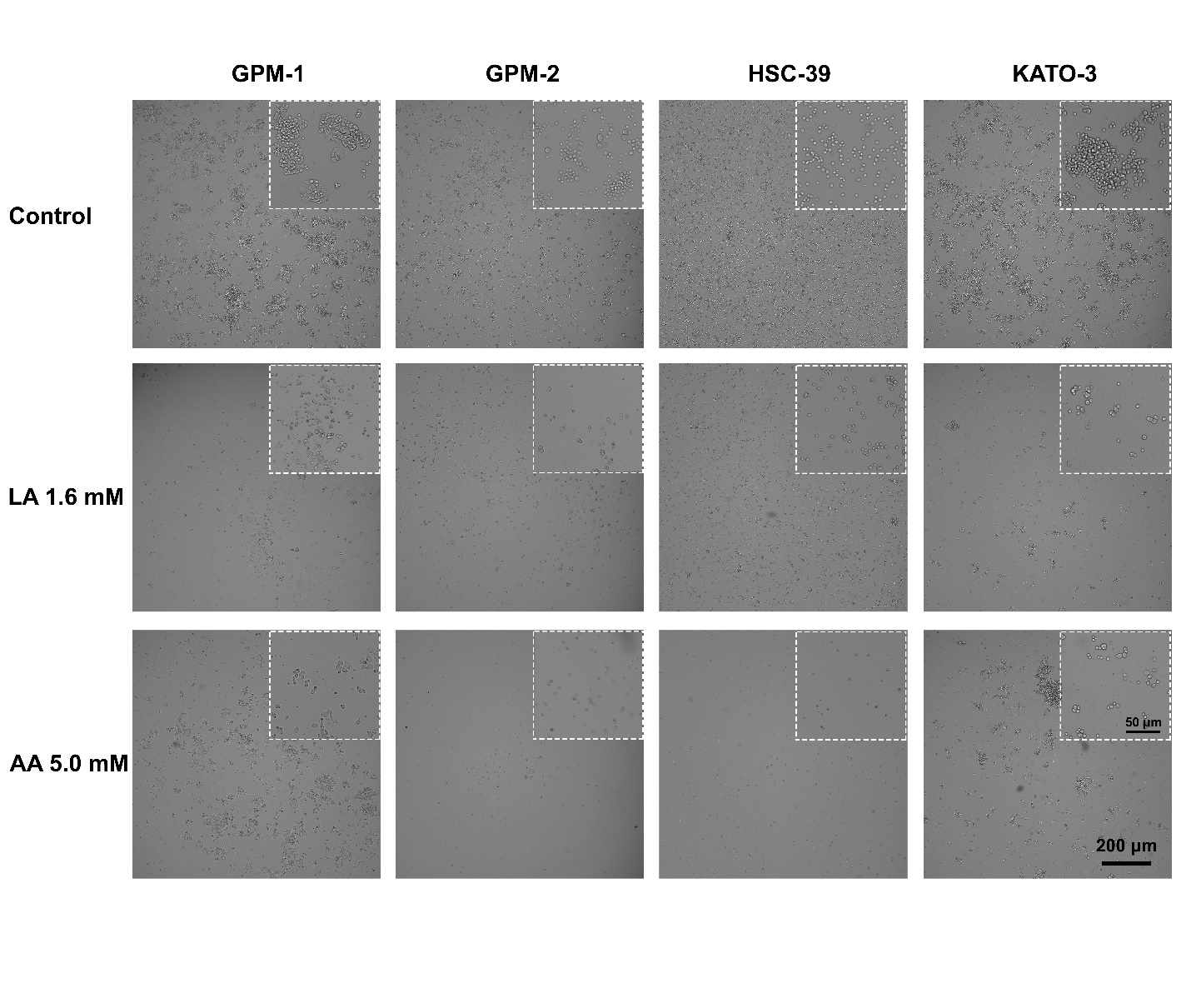


**Supplementary Fig. S3** Morphology of SRCC and Non-SRCC lines after LA (1.6 mM) and AA (5.0 mM) treatment for 16 hours. Scale bar is 50 and 200 um.


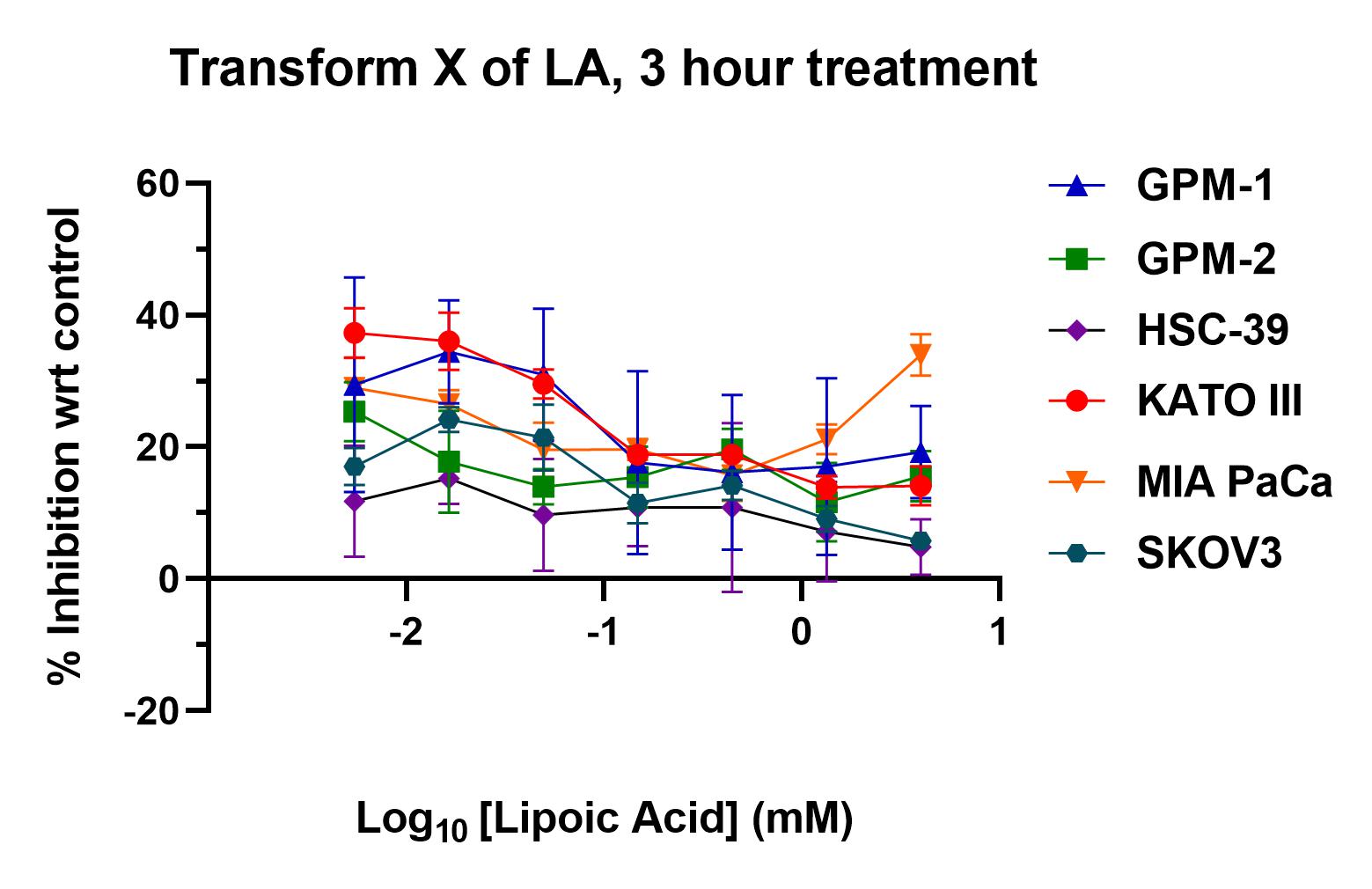


**Supplementary Fig. S4** Inhibition rate of LA on non-SRCC cells (GPM-1), patient-derived gastric origin SRCC (GPM-2), gastric-origin SRCCs (HSC-39 and KATO-3) and human pancreatic (MIA PaCa-2) and ovarian (SKOV-3) lines after 3-hour incubation.


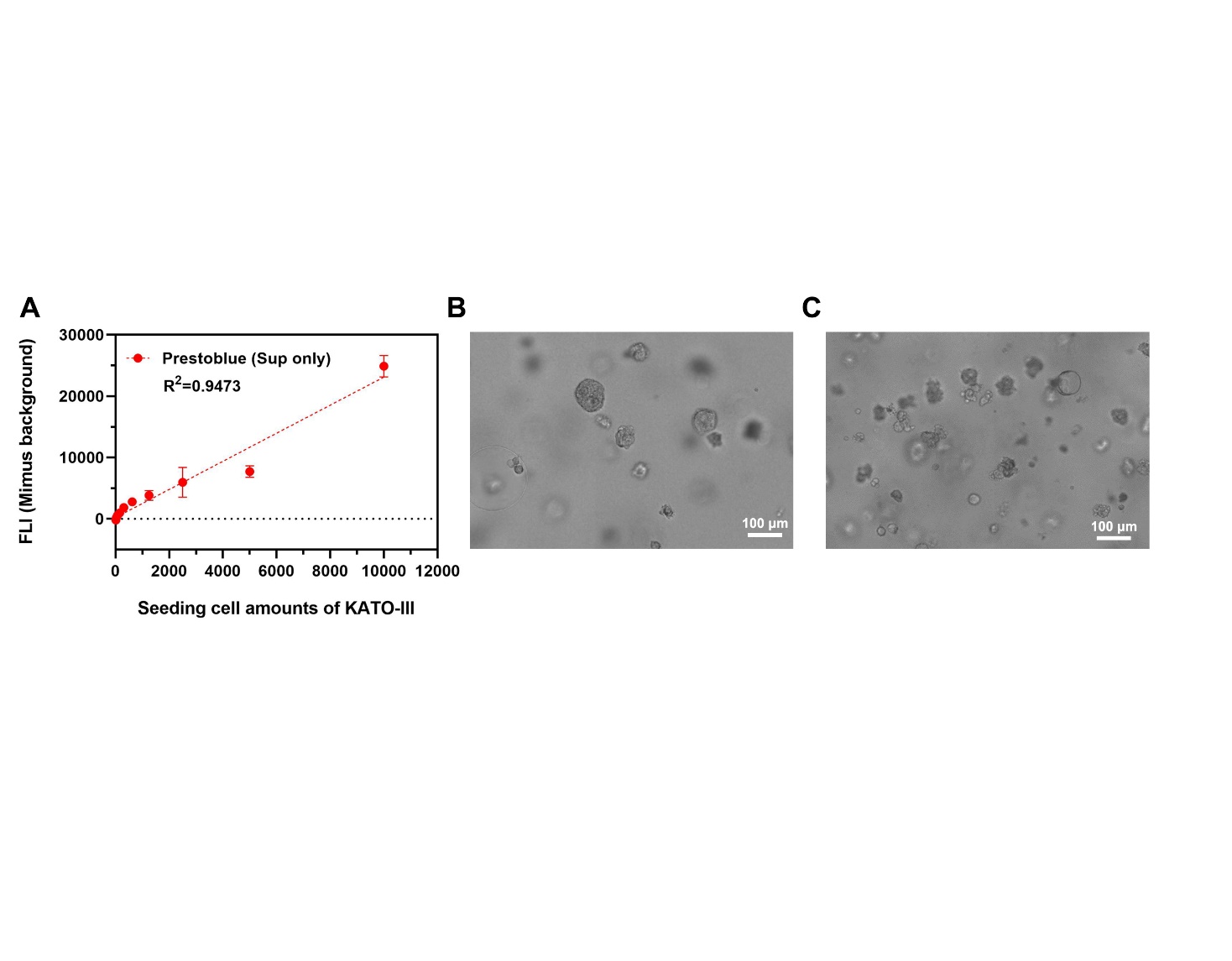


**Supplementary Fig. S5** Sensitivity of PrestoBlue in 3D-culture of KATO-3 cells. (A) The linear curve of PrestoBlue fluorescence signal (in supernatant only) and KATO-3 cell number. Optical images of 3D-cultured KATO-3 cells at cell density of (B) 10,000 and (C) 100,000 cells per well. Scale bar is 100 um.


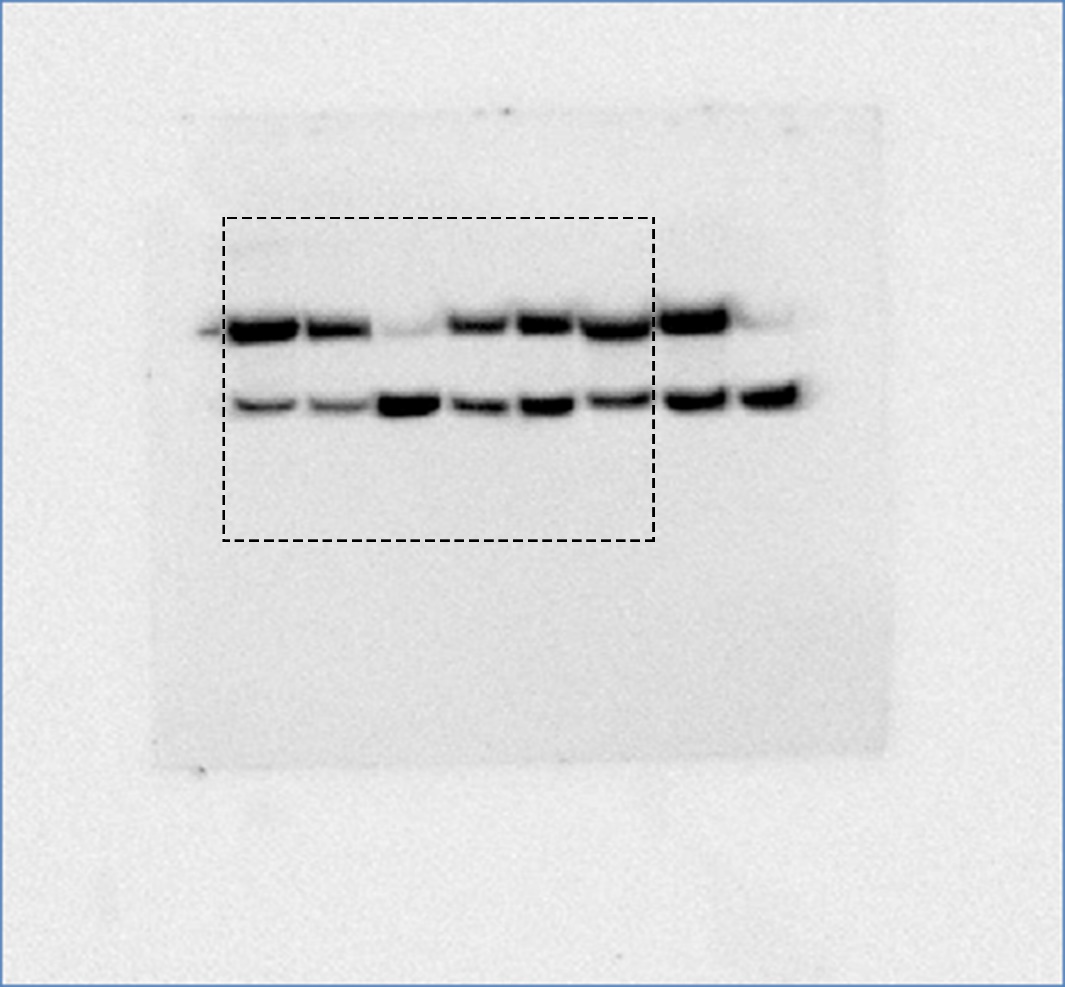


**Supplementary data of WB in Fig. 1** The original WB image of Fig. 1A. The top lane is Catalase and bottom lane is GAPDH. The dash line indicate the cropped area of Fig. 1A.
